# Supplementary material for: Biochar Obtained from Caryocar brasiliense Endocarp for Removal of Dyes from the Aqueous Medium
Source: Materials (Basel). 2022 Dec 19;15(24):9076. doi: 10.3390/ma15249076 (PMC9787617; doi:10.3390/ma15249076)
Supplement: Supplementary file 1 [file materials-15-09076-s001.zip › materials-1961654-supplementary.pdf]

materials

MDPI

Supplementary Materials

Biochar Obtained from *Caryocar brasiliense* Endocarp for Removal of Dyes from the Aqueous Medium

André L. F. C. Melo [1], Marcelo T. Carneiro [1], Ariane M. S. S. Nascimento [2], Alan I. S. Morais [2], Roosevelt D. S. Bezerra [3], Bartolomeu C. Viana [2], Josy A. Osajima [2] and Edson C. Silva-Filho [2,*]

1 Federal Institute of Piauí, Floriano Campus, Floriano 64808-475, PI, Brazil
2 Interdisciplinary Laboratory for Advanced Materials, Teresina 64049-550, PI, Brazil
3 Federal Institute of Piauí, Teresina-Central Campus, Teresina 64000-040, PI, Brazil
* Correspondence: edsonfilho@ufpi.edu.br

Supplementary Material

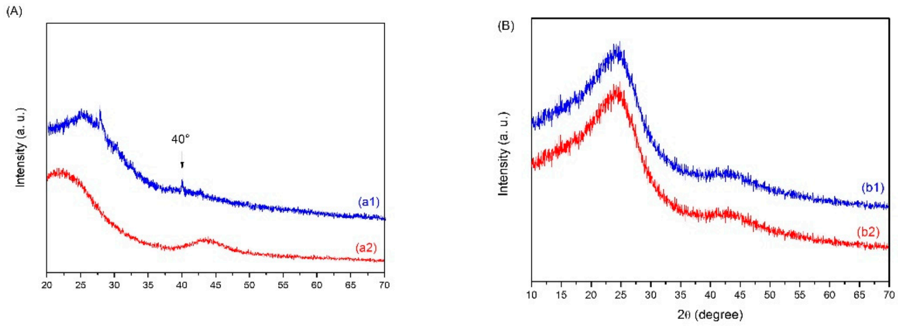

Figure S1. XRD for the studied samples of biochar of endocarp (BE) (a1) and activated biochar of the endocarp (ABE) (a2) (A); XRD pattern for the materials BE (b1) and ABE (b2) (B).

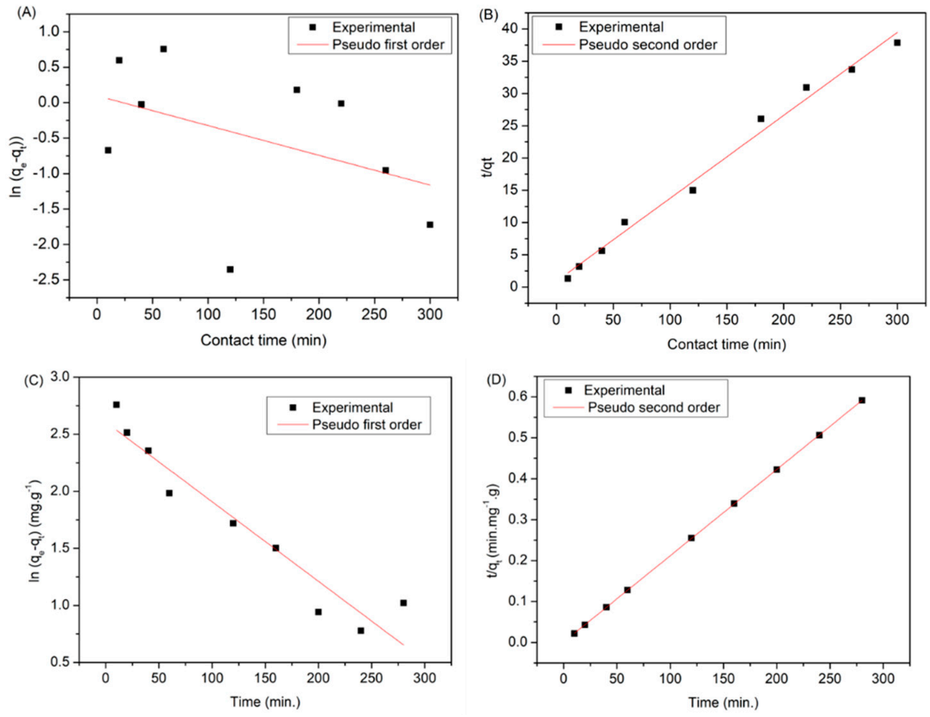

Figure S2. Adjustment to the kinetic model of pseudo-first-order (A) and pseudo-second-order (B) of biochar (BE); Fit to the kinetic model of pseudo-first-order (C) and pseudo-second-order (D) of biochar (ABE)

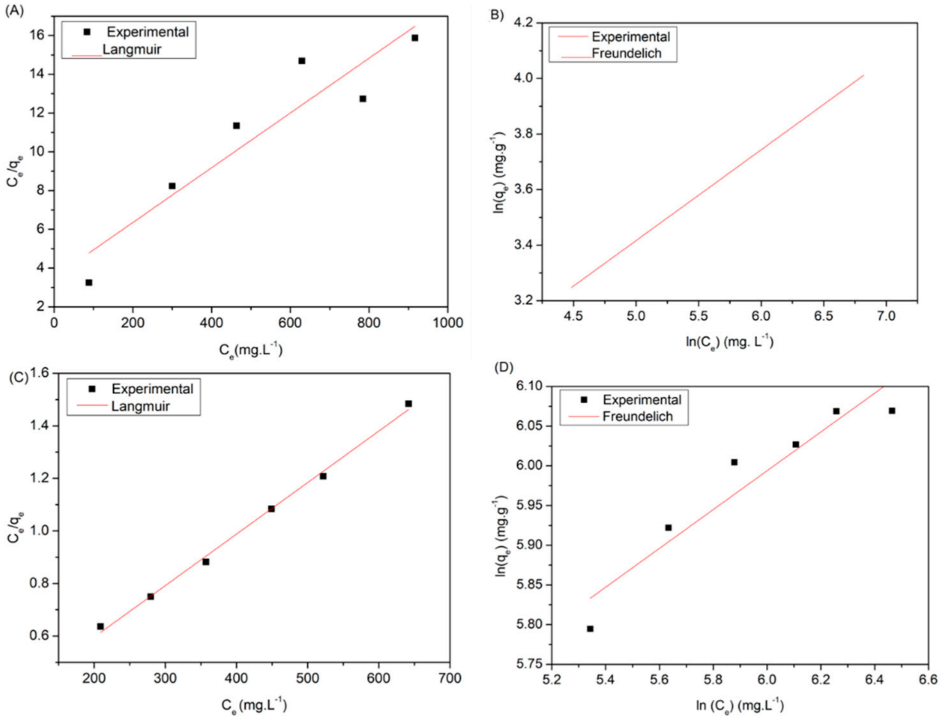

Figure S3. Adjustment to the isothermal model of Langmuir (A) and Freundlich (B) of biochar (BE); Fit to the isothermal model of biochar Langmuir (C) and Freundlich (D) (ABE).
